# Comparing cefotaxime and ceftriaxone in combating meningitis through nose-to-brain delivery using bio/chemoinformatics tools

**DOI:** 10.1038/s41598-020-78327-w

**Published:** 2020-12-04

**Authors:** Rania M. Hathout, Sherihan G. Abdelhamid, Ghadir S. El-Housseiny, Abdelkader A. Metwally

**Affiliations:** 1 Department of Pharmaceutics and Industrial Pharmacy, Faculty of Pharmacy, Ain Shams University, African Union Organization St., Cairo, 11566 Egypt; 2 Department of Biochemistry, Faculty of Pharmacy, Ain Shams University, Cairo, Egypt; 3 Department of Microbiology and Immunology, Faculty of Pharmacy, Ain Shams University, Cairo, Egypt; 4 Department of Pharmaceutics, Faculty of Pharmacy, Health Sciences Center, Kuwait University, Kuwait, Kuwait

**Keywords:** Computational biology and bioinformatics, Medical research, Nanoscience and technology

## Abstract

Bio/chemoinformatics tools can be deployed to compare antimicrobial agents aiming to select an efficient nose-to-brain formulation targeting the meningitis disease by utilizing the differences in the main structural, topological and electronic descriptors of the drugs. Cefotaxime and ceftriaxone were compared at the formulation level (by comparing the loading in gelatin and tripalmitin matrices as bases for the formation of nanoparticulate systems), at the biopharmaceutical level (through the interaction with mucin and the P-gp efflux pumps) and at the therapeutic level (through studying the interaction with *S. pneumoniae* bacterial receptors). GROMACS v4.6.5 software package was used to carry-out all-atom molecular dynamics simulations. Higher affinity of ceftriaxone was observed compared to cefotaxime on the investigated biopharmaceutical and therapeutic macromolecules. Both drugs showed successful docking on mucin, P-gp efflux pump and *S. pneumoniae* PBP1a and 2b; but ceftriaxone showed higher affinity to the P-gp efflux pump proteins and higher docking on mucin. Ceftriaxone showed less out-of-matrix diffusion and higher entrapment on the gelatin and the tripalmitin matrices. Accordingly, Ceftriaxone gelatin nanospheres or tripalmitin solid lipid nanoparticles may pose a more feasible and efficient nose-to-brain formulation targeting the meningitis disease compared to the cefotaxime counterparts.

## Introduction

Meningitis is a serious infection or inflammation of the meninges that can be caused by a wide variety of infectious agents^1^. Viruses account for up to half of cases while fungi (typically cryptococci) are less frequently detected, representing < 10% of cases^2^. Bacterial meningitis is considered the most severe form of this disease. The etiologic agents responsible for bacterial meningitis vary by age group. Among neonates, most cases are due to group B *Streptococcus agalactiae*, *Escherichia coli*, and *Listeria monocytogenes*. Although *Haemophilus (H.) influenzae* is implicated in bacterial meningitis in all age groups, it is predominant in children < 5 years of age^3^. However, the most common causes of bacterial meningitis are *Streptococcus (S.) pneumoniae* and *Neisseria (N.) meningitidis*, together accounting for approximately one-quarter of the cases. Pneumococcal meningitis is in general more common than meningococcal meningitis in children < 5 years of age and in the elderly (≥ 65 years of age), whereas meningococcal meningitis is more frequent among older children, adolescents and young adults^2,4^. Despite the existence of antibiotic therapies, acute bacterial meningitis causes significant morbidity and mortality. Survivors are prone to permanent consequences including brain damage, hearing loss, and learning disabilities^3^. Therefore, suspected cases should be treated with antibiotics as quickly as possible, even before the diagnosis can be confirmed, as a delay can result in a greater chance of adverse clinical outcomes.

The choice of antibiotic depends on the organism isolated. In most cases the initial treatment has to be empirical, but nonetheless based on epidemiological knowledge of the commonest organisms for each age group and local antibiotic resistance patterns^5^. Empirical antibiotic therapy should be bactericidal and achieve adequate cerebrospinal fluid (CSF) levels^4^. In most cases, a broad spectrum cephalosporin, especially cefotaxime (adults 2 g every 6 h; children 50 mg/kg every 6 h) or ceftriaxone (adults, 4 g/day; children, 50 mg/kg, to maximum 2 g/day single dose) is the most appropriate empirical choice. These cover *N. meningitides, S. pneumoniae, and H. influenzae* and penetrate CSF well^5^. International guidelines on the duration of treatment recommend 7–10-day treatment for *H. influenzae or N. meningitidis* meningitis and a 10–14-day treatment for *S. pneumoniae* meningitis^4^. Both ceftriaxone and cefotaxime are effective in the treatment of bacterial meningitis, but ceftriaxone has the advantage of being administered as a single daily dose^6^. Therefore, there is a need to compare the effectiveness of ceftriaxone and cefotaxime in the treatment of bacterial meningitis.

Cefotaxime (C_16_H_17_N_5_O_7_S_2_) and ceftriaxone (C_18_H_18_N_8_O_7_S_3_) are third generation cephalosporin antibiotics with broad spectrum bactericidal activity against Gram positive and Gram negative bacteria. They cross the blood–brain barrier (BBB) and reach therapeutic concentrations in the central nervous system (CNS). Their bactericidal activity results from the inhibition of cell wall synthesis via affinity for penicillin-binding proteins (PBPs). PBPs are membrane-anchored enzymes which participate in the terminal stages of assembling the bacterial cell wall, and in reshaping the cell wall during cell division. Inactivation of PBPs interferes with the cross-linkage of peptidoglycan chains necessary for bacterial cell wall strength and rigidity. This results in the weakening of the bacterial cell wall and causes cell lysis. (National Center for Biotechnology Information. PubChem Database. Ceftriaxone, CID = 5479530, Cefotaxime, CID = 5742673 https://pubchem.ncbi.nlm.nih.gov/compound (accessed on Dec. 16, 2019).

PBPs are targets for β-lactam antibiotics^7^. These proteins are classified based upon their molecular weights and conserved domain structures. Class A high-molecular-weight (HMW) PBPs are bifunctional proteins with transglycosylase (TG) and transpeptidase (TP) activities. They are believed to be the physiologically important enzymes that catalyze the final stages of peptidoglycan synthesis. Class B HMW PBPs are monofunctional TPs. Class C, or low-molecular-weight (LMW) PBPs are d,d-carboxypeptidases or d,d endopeptidases^8^. *S*. *pneumoniae*, possesses three class A PBPs (PBP1a, PBP1b, and PBP2a), two class B PBPs (PBP2x and PBP2b), and one class C PBP (PBP3) with d,d-carboxypeptidase activity^9,10^ and PBP2x possesses a C-terminal extension consisting of two PBP- and serine/threonine kinase-associated (PASTA) domains each containing one α-helix and three β-strands^11^.

The two class B PBPs (PBP2x and PBP2b) are individually essential in *S. pneumoniae*, performing critical roles in septal and peripheral peptidoglycan synthesis, respectively, and they are therefore likely critical targets for the β-lactam antibiotics^12^. β-Lactam antibiotics exert their antibacterial effect through covalent interactions with PBPs, thus blocking the terminal step in cell wall biosynthesis. β-Lactam resistance in *S. pneumoniae* is usually caused by amino acid substitutions in the penicillin-binding domains of 1 or more of its 6 PBPs, resulting from point mutations or mosaic genes following recombination^13^. Altered PBP1a, PBP2x and PBP2b are the most important PBPs for β-lactam resistance among clinical pneumococcal isolates^14,15^. Results of genetic and molecular analyses suggested that PBP2a contributed to cefotaxime resistance in some clinical isolates and laboratory strains of *S. pneumoniae*^16^*.*

For successful treatment of meningitis, effective antibiotic levels should be maintained in the brain all through the treatment period. Despite their great potency, systemic delivery of cephalosporins is associated with the potential risk of causing severe systemic side effects^17^. Therefore, there is demand for a patient compliant method to deliver cephalosporins to the brain. It is well known that there exists a direct nose-to-brain pathway via the olfactory region that could deliver drugs directly to the brain, bypassing the BBB. This route has been investigated as a potential route for delivery of several therapeutic agents via mechanisms that are still not clearly understood^18^. A key advantage of the nose-to-brain route is the possibility of reducing plasma exposure^19^, thus eliminating peripheral side effects. Intranasal delivery is also non-invasive, allows frequent administration and is less expensive than parenteral or oral therapy. Unlike parenteral formulations, nasal drops can be self-administered and do not require physician supervision during administration. Therefore, intranasal delivery of cephalosporins could be developed as a potential treatment approach for bacterial meningitis. The key factors that determine the efficacy of delivery via this route include the following: delivery to the olfactory area of the nose rather than the respiratory region, penetration enhancement of the active ingredient through the nasal epithelia, a longer retention time at the nasal mucosal surface, and a reduction in drug metabolism in the nasal cavity^20^.

Bioinformatics is an interdisciplinary science that represents the convergence of genomics, biotechnology, computer science and information technology^21^. It encompasses analysis and interpretation of data, modelling of biological phenomenon, development and implementation of computational algorithms and software tools in an effort to facilitate an understanding of the biological processes. Computational biology became increasingly important in various areas such as characterization of genes, determining structural and physiochemical properties of proteins, phylogenetic analyses, comparative or homology modeling, functional site location, characterization of active site for binding, docking of lead molecules into receptor binding sites, protein–protein interactions, molecular simulations, as well as drug designing^22^. Bioinformatics has several applications pertaining to pharmacy in the areas of drug discovery; designing and development; product/formulation designing; as well as pharmacokinetics and pharmacology^23^. Deploying genomics and proteomics, potential drug targets are identified by elucidating the interaction at the molecular level of a disease^24^. Molecular docking could predict the structure of intermolecular complex found between two molecules and predict the affinity between these biomolecules or receptors and potential drug candidate to find the best orientation of the ligand which would form a complex with overall minimum energy^25^.

In this study, a comparison between cefotaxime and ceftriaxone as third generation cephalosporins antibiotics against bacterial meningitis was performed at the formulation level (by comparing the loading in gelatin and tripalmitin matrices as bases for the formation of nanoparticulate systems), at the biopharmaceutical level (through the interaction with mucin and the P-gp efflux pumps) and at the therapeutic level (through studying the interaction with *S. pneumonia* bacterial receptors).

## Methodology

### Construction of the virtual carrier using molecular dynamics simulations

GROMACS^26^ v4.6.5 software package was used to carry-out all-atom molecular dynamics simulations. The atom typing and assignment of parameters and charges of the gelatin and tripalmitin matrices^27^ were carried-out online (https://cgenff.paramchem.org/) according to CHARMM general force field (CgenFF). To prepare the gelatin system, 48 peptide molecules were constructed, with 18 amino acids in each molecule. The primary sequence of the peptides was AGPRGQ(Hyp)GPAGPDGQ(Hyp)GP. On the other hand, the tripalmitin system contained 64 molecules of tripalmitin. The two matrices were then subjected to a molecular dynamics run, with full periodic boundary conditions, a time step of 2 fs, and a cut-off distance for Van der Waal’s and electrostatic interactions of 1 nm. PME was used to calculate electrostatic interactions and LINCS algorithm was used to constrain all bonds. The systems were equilibrated for 7 ns at 298 K using a v-rescale thermostat at a pressure of 1 bar using a Berendsen barostat.

### Obtaining the target peptides and proteins virtual matrices

The crystal structure of the relevant nose-to-brain delivery and the therapeutic targets related to the bacterial cell wall receptors were obtained from the protein data bank (http://www.rcsb.org). The following codes: 2ACM and 3G6I corresponded to Mucin and P-gp efflux-pump receptors, respectively. 26CW and 2WAD corresponded to the protein binding proteins 1A and 2B, respectively. The polar hydrogens were added to the obtained pdb files using MOE version 2014.0901 (Chemical Computing Group Inc., Montreal, Canada).

### Preparing the drugs chemical structures for docking

The isomeric SMILES corresponding to the chemical structures of the studied antibiotics; cefotaxime and ceftriaxone were obtained using PubChem. The corresponding 3D chemical structures were generated using the builder function of MOE version 2014.0901 (Chemical Computing Group Inc., Montreal, Canada). Further, energy minimization was carried out for all the investigated molecules using MMFF94x forcefield of the same software^28,29^.

### Docking of the investigated drugs on the investigated carrier

The docking analysis was employed using MOE version 2014.0901 (Chemical Computing Group Inc., Montreal, Canada). The pdb file of the protein nanoparticles matrix was imported to MOE where the identification of the binding site was performed using MOE's “Site finder” tool^30^ to be ready for docking using the "*triangle matcher*” as a placement method.

This software creates dummy atoms around the docking target atoms. These dummy atoms are considered the docking positions. The London ΔG and ASE scores were utilized for calculating the binding energies scoring values. The London ΔG scoring function estimates the free energy of binding of the ligand from a given pose. The functional form is a sum of terms:

$$ \Delta G = c + E_{flex} + \sum\limits_{{h{ - }bonds}} {C_{HB} f_{HB} } + \sum\limits_{{m{ - }lig}} {C_{M} f_{M} } + \sum\limits_{atoms\;i} {\Delta D_{i} } $$

where *c* represents the average gain/loss of rotational and translational entropy; *E*_*flex*_ is the energy due to the loss of flexibility of the ligand (calculated from ligand topology only); *f*_*HB*_ measures geometric imperfections of hydrogen bonds and takes a value in [0,1]; *c*_*HB*_ is the energy of an ideal hydrogen bond; *f*_*M*_ measures geometric imperfections of metal ligations and takes a value in [0,1]; *c*_*M*_ is the energy of an ideal metal ligation; and *D*_*i*_ is the desolvation energy of atom *i*. The difference in desolvation energies is calculated according to the formula

$$ \Delta D_{i} = c_{i} R_{i}^{3} \left\{ {\iiint\limits_{u \notin A \cup B} {|u|^{ - 6} du} - \iiint\limits_{u \notin B} {|u|^{ - 6} du}} \right\} $$

where *A* and *B* are the protein and/or ligand volumes with atom *i* belonging to volume *B*; *R*_*i*_ is the solvation radius of atom *i* (taken as the OPLS-AA van der Waals sigma parameter plus 0.5 Å); and *c*_*i*_ is the desolvation coefficient of atom *i*. The coefficients {*c*, *c*_*HB*_, *c*_*M*_, *c*_*i*_} were fitted from ~ 400 X-ray crystal structures of protein–ligand complexes with available experimental pKi data. Atoms are categorized into about a dozen atom types for the assignment of the *c*_*i*_ coefficients. The triple integrals are approximated using Generalized Born integral formulas. Like all commonly used scoring functions, lower binding energies (ΔG, kcal/mole) scores indicate more favourable interactions.

### Calculating the main descriptors of the investigated drugs

In order to explain the differences in docking scores observed for the studied drugs, some crucial constitutional, electronic and topological descriptors were calculated for the studied drugs. The selected descriptors were the molecular weight, xLogP, topological polar surface area, number of H-atoms donors and acceptors and finally the fragment complexity. The descriptors were calculated using *Bioclipse *version 2.6 (Bioclipse project, Uppsala University, Sweden) using the molecules mol files generated using Chem3D Ultra version 10 (Cambridgesoft, Perkin Elmer, Akron, Ohio).

## Results and discussion

The gelatin and tripalmitin have been rationally selected as the nanoparticulate matrices material for loading the investigated drugs due to their proven successful compatibility with the olfactory nerves and regions, their successful penetration through the drug brain barrier and their efficiency (especially gelatin) on loading several hydrophilic and hydrophobic drugs^31–33^. The successful construction of the gelatin and tripalmitin virtual matrices using the adopted molecular dynamics method was obtained after following the same protocols of the authors previous studies^34–37^. Table 1 demonstrates the results of docking the investigated drugs; cefotaxime and ceftriaxone on the selected carriers. The higher score of ceftriaxone on the gelatin matrix could be attributed to its more hydrophilic nature demonstrated by its lower Log P (− 1.59), higher total polar surface area (506.48) and higher number of h-bond donors and acceptors (5 and 9, respectively) that can interact with the gelatin carboxylic and amino groups^38,39^ compared to the cefotaxime counterparts. Table 2 depicts the SMILES (that were needed for docking experiments, electronic, constitutional and topological physico-chemical descriptors) of the two studied antibiotics. Moreover, the less molecular flexibility and higher molecular weight of ceftriaxone lead to less out-of-matrix diffusion and higher entrapment of this molecule whether on the gelatin or the tripalmitin matrices^40–42^. Therefore, these factors would play a very important role in deciding the best drug–carrier pair^43^.

**Table 1:** Docking binding energy (ΔG) values after docking of the investigated drugs on the nose-to-brain related macromolecules.

Macromolecule (carrier/protein)—PDB code	Binding energy (kcal/mole)	
	Cefotaxime	Ceftriaxone
Gelatin matrix (gelatin nanospheres)	− 8.53 ± 0.4	− 11.68 ± 0.5
Tripalmitin matrix (solid lipid nanoparticles)	− 9.60 ± 0.2	− 10.77 ± 0.4
Mucin—2ACM	− 11.67 ± 0.2	− 11.70 ± 0.2
P-gp efflux pump—3G61	− 8.95 ± 0.3	− 10.22 ± 0.4
Protein binding protein 1A (PBP-1A) 26CW	− 12.32 ± 0.1	− 13.18 ± 0.2
Protein binding protein 2B (PBP-2B) 2WAD	− 12.11 ± 0.2	− 14.35 ± 0.03

**Table 2:** Main physico-chemical descriptors of the investigated drugs.

Molecule	SMILES	Total polar surface area	Number of H-bond acceptors	Number of H-bond donors	Molecular flexibility	logP (o/w)	Molecular weight
Cefotaxime	s1cc(nc1N)C(= NOC)C(= O)NC1C2SCC(COC(= O)C) = C(N2C1 = O)C(O) = O	381.41	7	4	5.95	− 0.64	455.472
Ceftriaxone	s1cc(nc1N)C(= NOC)C(= O)NC1C2SCC(CSC3 = NC(= O)C(= O)NN3C) = C(N2C1 = O)C(O) = O	506.48	9	5	− 1.161	− 1.59	554.589

**Figure 1:**
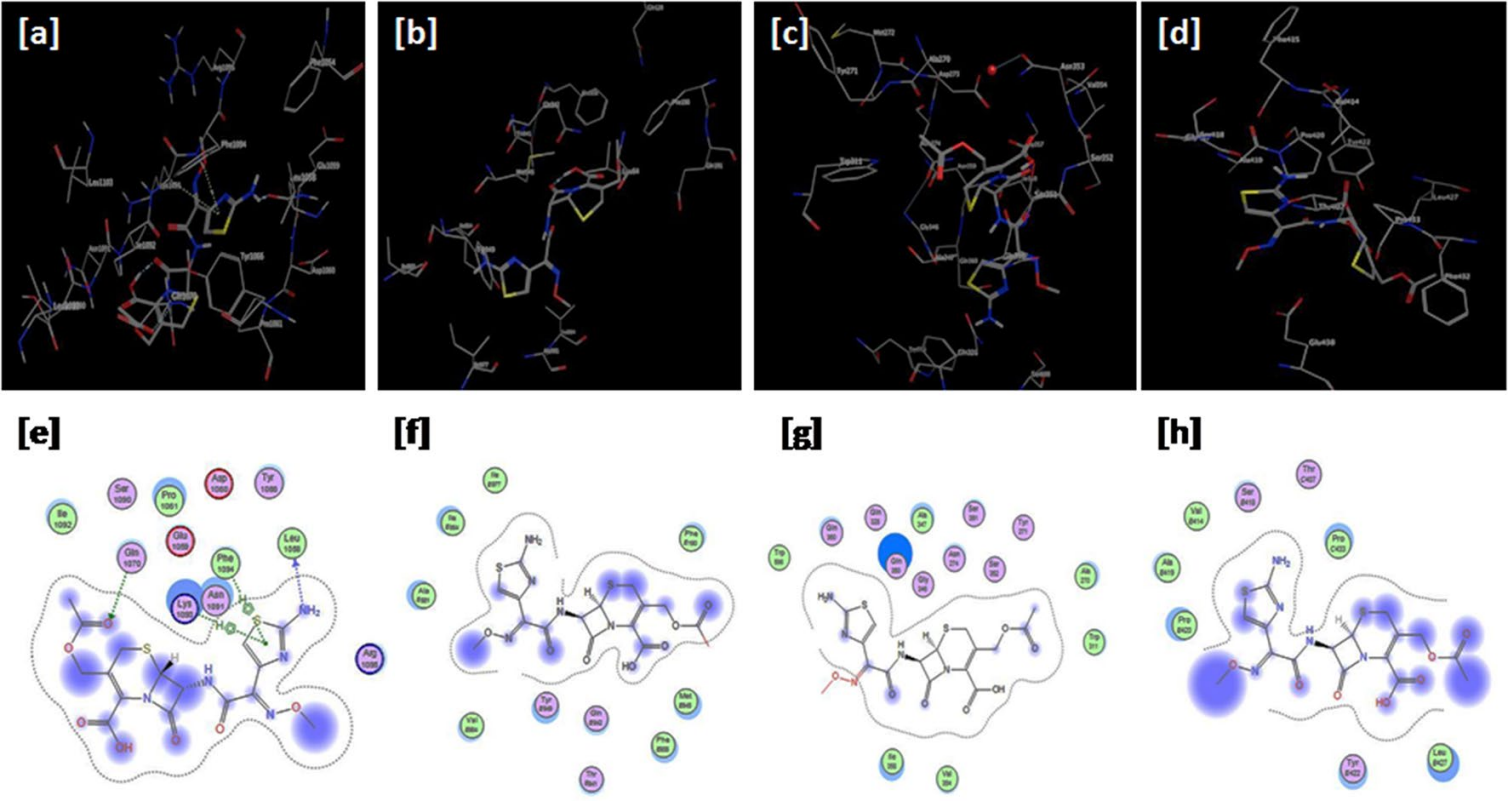
Docking results of cefotaxime on (**a**–**e**) 2ACM, (**b**–**f**) 3G61, (**c**–**g**) 2C6W and (**d**–**h**) 2WAD. Upper and lower panels represent 3D and 2D images, respectively.

**Figure 2:**
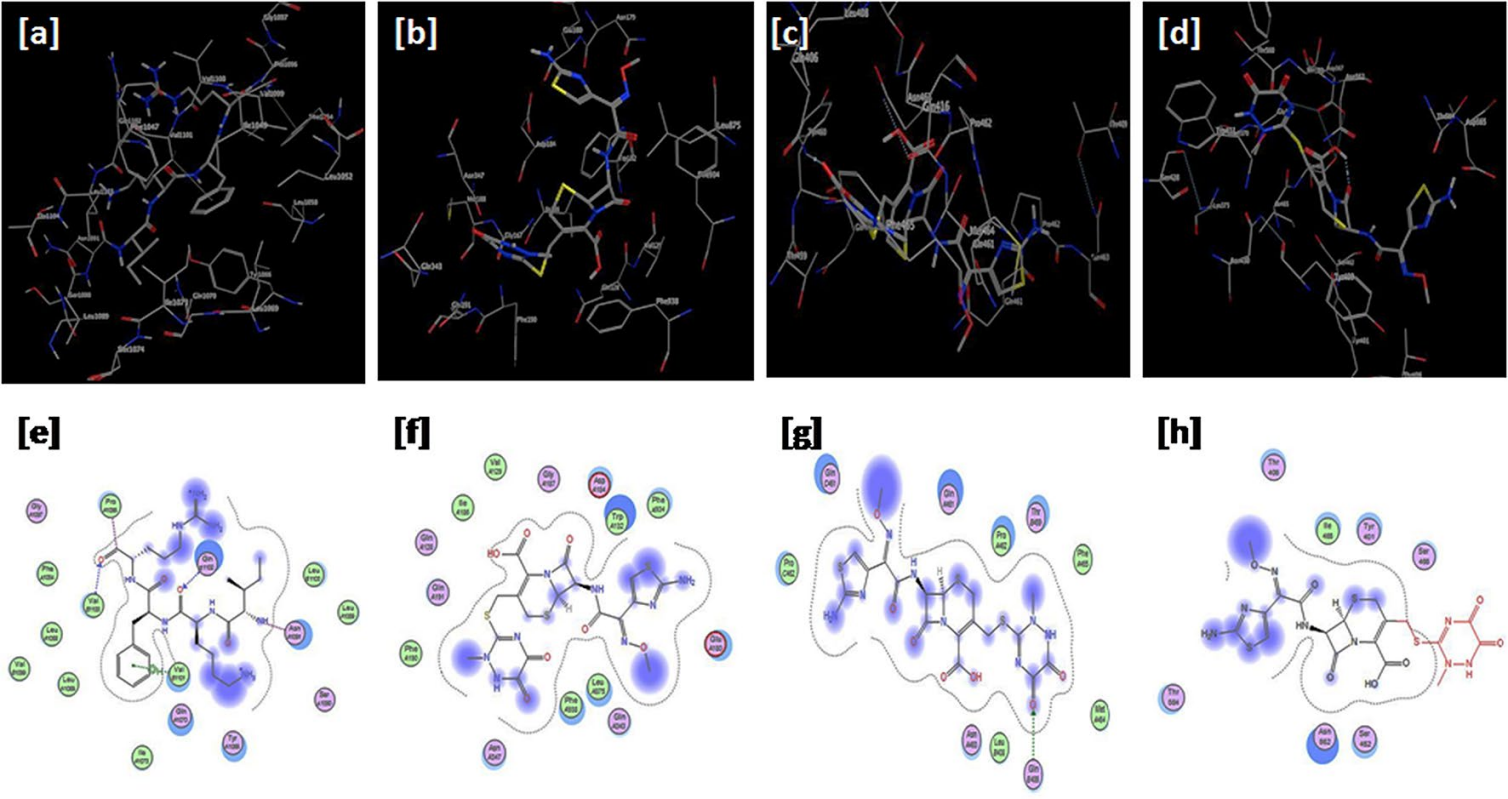
Docking results of ceftriaxone on (**a**–**e**) 2ACM, (**b**–**f**) 3G61, (**c**–**g**) 2C6W and (**d**–**h**) 2WAD. Upper and lower panels represent 3D and 2D images, respectively.

Figure 1 also depicts the successful docking of cefotaxime on the macromolecules viz*.* the proteins and peptides relevant to the brain delivery (e.g. mucin and P-gp efflux pump) or the meningitis disease bacterial therapeutic targets (e.g. *S. pneumoniae* PBPa and 2b). Figure 2 demonstrates the successful docking of ceftriaxone on the same targets.

Table 1 shows the obtained binding energies after docking of the two antibiotics on the investigated biopharmaceutical and therapeutic macromolecules. The recorded results showed the higher affinity of ceftriaxone compared to cefotaxime. On the delivery level, the docking results of the two drugs on mucin as an indicator of the ability of the antibiotic for better mucoadhesion in the olfactory region was in favor of ceftriaxone. Furthermore, higher affinity of the ceftriaxone to the P-gp efflux pump proteins (responsible for the expulsion of drugs outside the brain cells and the blood–brain barrier) was observed as revealed by the lower binding energy scores. This finding warrants the usage of a carrier system such as gelatin nanospheres that can circumvent this usual biopharmaceutical hindrance. The interpretation of these results can also be ascribed to the differences in the main structural, topological and electronic descriptors of the two investigated drugs (Table 2) as previously discussed by Metwally and Hathout^36,44^.

Recently, the authors have proven the contribution of these main descriptors of drugs on the affinity and hence the loading of drugs on different carriers (Tripalmitin solid lipid nanoparticles, protein and PLGA nanoparticles)^36^ where the drug molecular weight, total polar surface area, and molecular flexibility have shown great influences of the drugs to different proteins and receptors^40,45^.

It should be noticed that the difference in the number of hydrogen bond donors and acceptors between the two closely related chemical structures should have contributed to the superior interaction of ceftriaxone over cefotaxime with the investigated bacterial proteins indicating more potency.

The obtained molecular docking experiments results on the selected biological protein receptors may explain the effectiveness of both drugs in meningitis treatment at clinical studies but in less doses and dosing frequency in case of ceftriaxone. In a well-conducted clinical study on 82 children, the effectiveness and safety of the two-antibiotics were evaluated in the short-term treatment of primary bacterial meningitis using a prospective, randomized, multicenter study design. Ceftriaxone was effective at a single dose (100 mg/kg on the first day followed by 75 mg/kg/day) while cefotaxime needed four divided doses (200 mg/kg/day) per day for 4–7 days^6^.

In light of the obtained results, it can be concluded that the ceftriaxone nose-to-brain delivery should pose a more feasible and efficient therapy to the *S. pneumoniae* related meningitis disease as compared to cefotaxime.

## Conclusion and future perspective

In the current work, the use of several bio/chemoinformatics tools have proven that ceftriaxone gelatin nanospheres or tripalmitin solid lipid nanoparticles may pose better nose-to-brain formulation targeting the meningitis disease compared to the cefotaxime counterparts. The current study could find a comprehensive solution to the usual debate about the feasibility of performing extensive researches on anti-microbials and/or biosimilars aiming for better alternatives considering all aspects and points of views; formulation, biopharmaceutical or therapeutic. This kind of research would also offer a new platform in medicines design which can save formulators and pharmacists huge time and resources spent on wet-lab experimentation. Moreover, the probability of errors and inaccurate results obtained from biological experiments are relatively reduced.
